# Linear B Cell Epitopes Derived from the Multifunctional Surface Lipoprotein BBK32 as Targets for the Serodiagnosis of Lyme Disease

**DOI:** 10.1128/mSphere.00111-19

**Published:** 2019-05-01

**Authors:** Christina Toumanios, Lauren Prisco, Raymond J. Dattwyler, Paul M. Arnaboldi

**Affiliations:** a Summer Trainees in Academic Research Program, Graduate School of Basic Medical Sciences, New York Medical College, Valhalla, New York, USA; b Colgate University, Hamilton, New York, USA; c Cornell University, Ithaca, New York, USA; d Biopeptides Corp., East Setauket, New York, USA; e Department of Microbiology and Immunology, New York Medical College, Valhalla, New York, USA; UMKC School of Medicine

**Keywords:** BBK32, *Borrelia burgdorferi*, Lyme disease, serology

## Abstract

BBK32 is a multifunctional surface lipoprotein expressed by Borrelia burgdorferi *sensu lato,* the causative agent of Lyme disease. Previous studies suggested that BBK32 could be a sensitive antigen target of new, more effective, serodiagnostic assays for the laboratory diagnosis of Lyme disease. However, nonspecific antibody binding to full-length BBK32 has hampered its use as a target in clinical assays. Specificity can be improved by the use of peptides composed of linear B cell epitopes that are unique to B. burgdorferi, eliminating cross-reactive epitopes that bind to antibodies generated by non-B. burgdorferi antigens. In this study, we identified linear B cell epitopes in 2 regions, BBK32 amino acids 16 to 30 [BBK32(16–30)] and BBK32 amino acids 51 to 80 [BBK32(51–80)], by probing overlapping peptide libraries of BBK32 with serum from patients with early Lyme disease. We screened synthetic peptides containing these epitopes using a large panel of serum (*n = *355) obtained from patients with erythema migrans lesions (early Lyme disease), Lyme arthritis, syphilis, rheumatoid arthritis, or healthy volunteers. BBK32(16–30) demonstrated a nearly universal antibody binding in serum from all patients, indicating that regions of BBK32 are highly cross-reactive. BBK32(51–80) was less cross-reactive, being able to distinguish serum from Lyme disease patients from control patient serum; however, an unacceptable level of antibody binding was still observed in control samples, resulting in a reduced specificity (94.7%). These results indicate that BBK32 contains cross-reactive epitopes that make it a poor antigen target for inclusion in a serodiagnostic assay for Lyme disease and highlight the difficulties in identifying highly sensitive and specific seroassay targets.

**IMPORTANCE** Lyme disease is an infectious disease that has the potential to cause significant morbidity with damage to nervous and musculoskeletal systems if left untreated. Appropriate antibiotic treatment during early infection prevents disease progression. Unfortunately, currently available diagnostics are suboptimal in the detection of early disease. The inability to confirm Borrelia infection using laboratory methods during early disease is, in part, responsible for much of the controversy surrounding Lyme disease today. As a result, there has been significant investment in the identification of new antigen targets to generate diagnostic assays that are more sensitive for the detection of early infection. The importance of our research is that in our evaluation of BBK32, an antigen that was previously identified as a promising target for use in serodiagnostics, we found a high degree of cross-reactivity that could compromise the specificity of assays that utilize this antigen, leading to false-positive diagnoses.

## Abstract (precis)

Lyme disease is an infectious disease that has the potential to cause significant morbidity with damage to nervous and musculoskeletal systems if left untreated. Appropriate antibiotic treatment during early infection prevents disease progression. Unfortunately, currently available diagnostics are suboptimal in the detection of early disease. The inability to confirm Borrelia infection using laboratory methods during early disease is, in part, responsible for much of the controversy surrounding Lyme disease today. As a result, there has been significant investment in the identification of new antigen targets to generate diagnostic assays that are more sensitive for the detection of early infection. The importance of our research is that in our evaluation of BBK32, an antigen that was previously identified as a promising target for use in serodiagnostics, we found a high degree of cross-reactivity that could compromise the specificity of assays that utilize this antigen, leading to false-positive diagnoses.

## INTRODUCTION

The laboratory diagnosis of Lyme disease is based on the detection of serum antibodies against Borrelia burgdorferi, the principal causative agent of the disease in the United States (1). In 1995, the Centers for Disease Control and Prevention recommended the use of a two-tier paradigm for determining seropositivity, where the first tier consists of a sensitive enzyme immunoassay (EIA) or immunofluorescence assay (IFA) which, if positive or equivocal, is followed by a second-tier Western blot analysis (2). The two-tier paradigm was put in place to deal with nonspecificity associated with first-tier assays and remains in place today (3–7). The second-tier Western blot analysis is qualitative and subjectively scored by comparing band intensity to a control; it requires 2/3 specific bands or 5/10 specific bands to be considered positive for IgM or IgG blots, respectively (1, 2). Though the two-tier paradigm increased the specificity of serodiagnosis, during early disease (when the antibody response is developing), the sensitivity of the assay is unacceptably low (less than 50%) (3–7). The nonspecificity of first-tier assays is primarily the result of using whole bacterial lysates or whole bacterial proteins as antigen targets (8, 9). These antigens contain a mixture of epitopes, with some that are unique to B. burgdorferi and some that are conserved and are present in other antigens (8–11). Synthetic peptides containing epitopes that are unique to B. burgdorferi can be used as assay targets as a method to reduce nonspecificity, thus improving the specificity of first-tier assays (10, 12–14). Improved specificity could lead to the elimination of the need for the second-tier Western blot analysis. Eliminating the second-tier Western blot analysis would allow for greater sensitivity of Lyme disease serodiagnostics, especially in the early stages of infection. For this approach to be effective, epitope mapping of multiple Lyme disease antigens must be performed to identify unique peptides capable of serving as sensitive and specific serodiagnostic targets.

BBK32 is a multifunctional surface-expressed lipoprotein produced by B. burgdorferi in engorged ticks and throughout the different stages of mammalian infection (15). It binds to fibronectin and glycosaminoglycans, as well as the complement protein C1, and may play a role in the inhibition of complement activation, bacterial dissemination, and joint colonization (16–21). Since it is expressed in the feeding tick and throughout mammalian infection (15), BBK32 has been investigated as an antigen target for serodiagnostic laboratory assays (22–26). Early studies suggested that BBK32 could have value as a serodiagnostic target (22–25), with reported sensitivities as high as 87% for the positive detection of anti-*Borrelia* IgG antibodies in patients with early Lyme disease (23). However, unacceptable levels of nonspecific antibody binding to whole BBK32 protein in control populations were observed (26). The use of protein fragments rather than whole protein decreased background antibody binding in healthy control populations, reducing cutoff values for positivity and improving assay sensitivity (26). Despite this, specificity (antibody binding in control specimens) remained a problem (26). In the present study, we performed epitope mapping of whole BBK32, identifying several linear B cell epitopes. We evaluated these epitopes for sensitivity and specificity as antigen targets in a serodiagnostic assay for Lyme disease, using large panels of serum from patients with well-defined early and late Lyme disease, control serum from healthy volunteers residing in areas of endemicity and nonendemicity for Lyme disease, and from individuals with illnesses that are associated with the production of cross-reactive antibody.

## RESULTS

**FIG 1:**
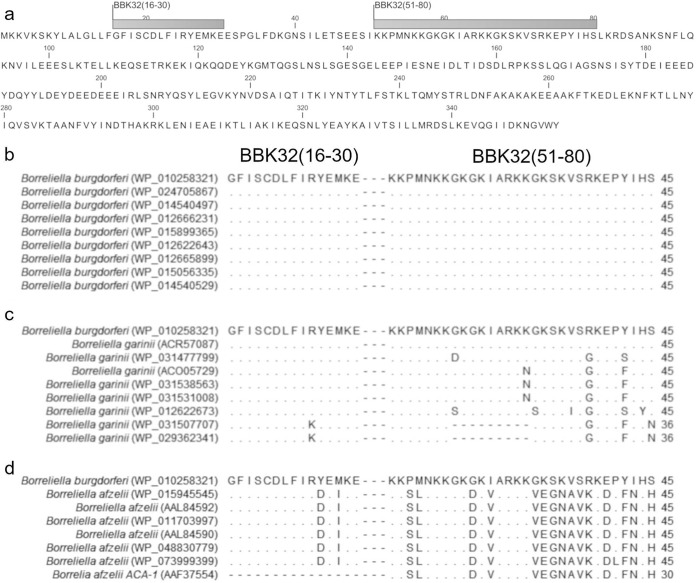
Sequence analysis of linear epitopes of BBK32 identified by linear B cell epitope mapping. (**a)** Illustration depicting the location of the two epitope regions in the full BBK32 protein of the B. burgdorferi B31 strain. (b to d) Sequence alignments of BBK32(16–30) and BBK32(51–80) between the B. burgdorferi B31 sequence used for epitope mapping (top row) and comparable amino acid sequences in different strains of B. burgdorferi (b), B. afzelii (c), and B. garinii (d). Dots indicate the presence of identical amino acid at that position, dashes indicate that the amino acid at that position is missing, and letters indicate that a different amino acid is present at that position. The sequence identifier (ID) is the Latin name (NCBI RefSeq accession no.).

Linear B cell epitope mapping of BBK32 revealed two regions of interest, BBK32 amino acids 16 to 30 [BBK32(16–30)] and BBK32 amino acids 51 to 80 [BBK32(51–80)] (Fig. 1a). The 15-amino acid (aa) peptide corresponding to the sequence of BBK32(16–30) was bound by antibody in all 8 patient serum samples used for screening, and it returned the highest binding signals out of any peptide in the array (data not shown). Antibody binding was not observed in any patient serum sample for the peptides on either side of BBK32(16–30), indicating that the epitope was entirely contained within the 15-aa sequence. The 30-aa region, BBK32(51–80), included four overlapping peptides [BBK32(51–65), BBK32(56–70), BBK32 (61–75), and BBK32(66–80)]. Antibody bound to BBK32(51–65) and BBK32(66–80) in 7 of 8 patient serum samples, while antibody bound to BBK32(56–70) and BBK32(61–75) in all 8 patient serum samples (data not shown). Antibody binding was observed in only one patient sample for the peptides on either side of the BBK32(51–80) region.

We aligned the sequences identified in the epitope mapping with available database sequences from B. burgdorferi, B. afzelii, and B. garinii, the three principal Lyme disease-causing species of Borrelia, using the protein Basic Local Alignment Search Tool (pBLAST) at the NCBI (27). B. burgdorferi is the principal causative agent of Lyme disease in North America, while all three species cause Lyme disease in Europe. Lyme disease in the United States is also caused by Borrelia mayonii; however, no putative BBK32 sequence for B. mayonii has been identified, and alignments attempted using B. mayonii as a target identified no homologous sequences. As indicated in Fig. 1b to d, the sequence of BBK32(16–30) is highly conserved across the three species, with a 2-aa difference among some strains of B. afzelii and a 1-aa difference among available strains of B. garinii. The sequence was fully conserved among different strains of B. burgdorferi. On the other hand, the sequence for the 30-aa region corresponding to BBK32(51–80) was primarily conserved within each species but had substantial variability between the different species (Fig. 1b to d). B. garinii had the most within-species sequence, variation with some B. garinii strains having a 9-aa deletion in the middle of the sequence, while other strains had 3 or more single-amino-acid substitutions (Fig. 1d). BBK32(51–80) was highly conserved within species for both B. burgdorferi and B. afzelii (Fig. 1b and c). At the same time, the greatest sequence difference between species was between B. burgdorferi and B. afzelii, with at least 50% of the amino acids of BBK32(51–80) differing between the two species. This suggests that the sequence of BBK32(51–80) derived from the B31 strain of B. burgdorferi could have limited utility as a diagnostic target in regions where B. afzelii is present.

**FIG 2:**
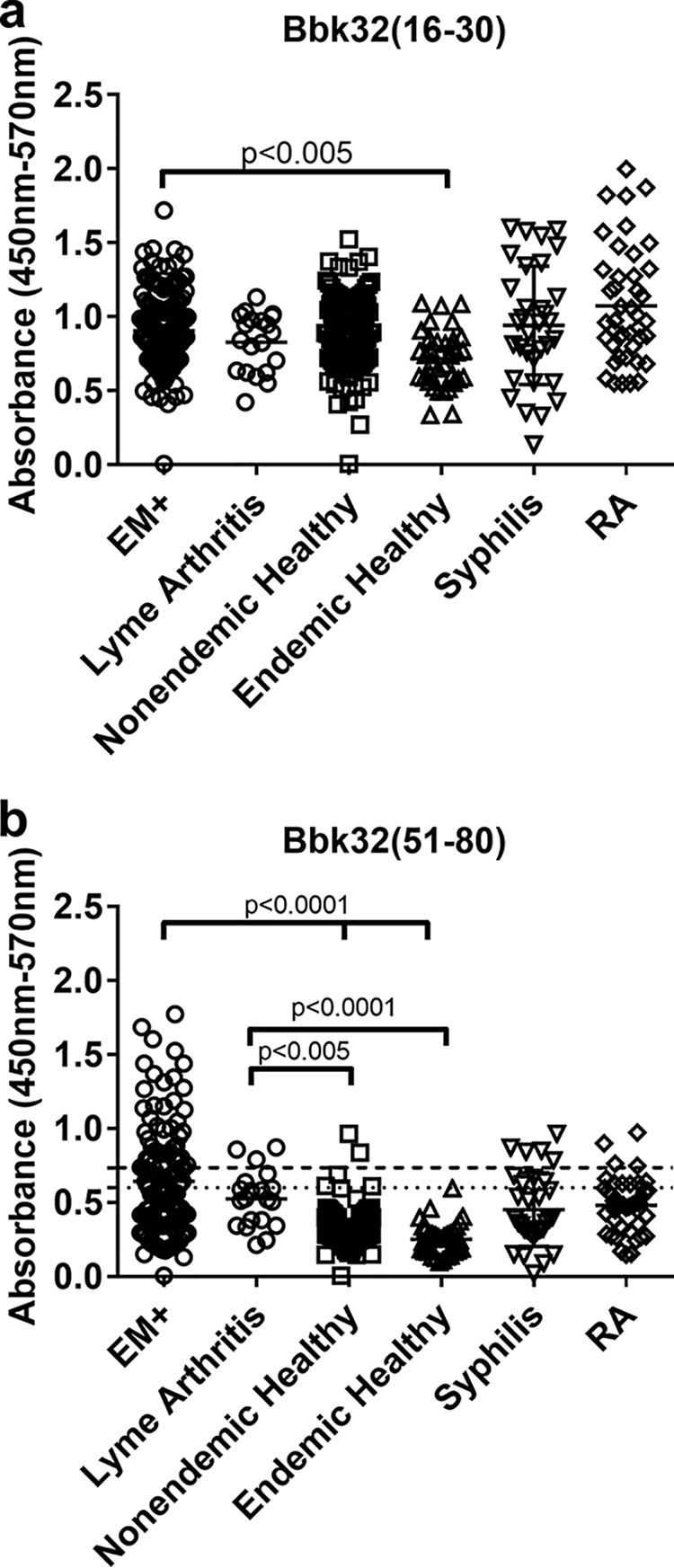
(a and b) Antibody binding to BBK32(16–30) (a) or BBK32(51–80) (b) in serum samples from patients with early Lyme disease (erythema migrans [EM+]), Lyme arthritis, healthy controls from patients living in regions not endemic or endemic for Lyme disease (nonendemic normal and endemic normal, respectively), patients with syphilis, or patients with rheumatoid arthritis. The dashed line represents the cutoff for positive binding, 3SD from the mean (horizontal solid bar) of the healthy controls. The dotted line represents the cutoff for equivocal binding, 2SD from the mean of healthy controls. Antibody binding was measured by ELISA and the results presented as absorbance at nm 450 to reference absorbance at 570 nm. Total sample numbers (*n*) are indicated in Table 1. Comparisons were performed for all groups, and only differences that generated a *P* value of less than <0.05 are shown. All other comparisons are not significant.

Synthetic peptides were generated corresponding to the identified sequences, BBK32(16–30) and BBK32(51–80). We evaluated serum antibody binding to peptides using a standard enzyme-linked immunosorbent assay (ELISA) and the panels of sera described below in Materials and Methods. Positive binding was defined as an absorbance greater than 3 standard deviations (3SD) from the mean absorbance of antibody binding to peptide in serum samples from healthy controls in areas of nonendemicity (nonendemic healthy control). Equivocal binding was defined as an absorbance between 2SD and 3SD from the mean absorbance of antibody binding to peptide in nonendemic healthy control serum, and negative binding was less than 2SD from the mean of nonendemic healthy controls. We detected similar antibody binding to BBK32(16–32) in all serum sets regardless of source, indicating that the peptide sequence was universally cross-reactive (Fig. 2a). Apart from the group of healthy controls in areas of endemicity (endemic healthy control group), the mean absorbance of antibody binding was within one SD for all groups. Mean antibody binding in the endemic healthy control group demonstrated a mathematically significant difference (*P* < 0.005) compared to erythema migrans-positive (EM+) patients; however, the biological relevance of this difference is questionable (Fig. 2a). Given the substantial nonspecificity observed, we did not calculate positive antibody binding for BBK32(16–30).

BBK32(51–80) demonstrated a substantially different antibody binding profile from that of BBK32(16–30). Antibody binding absorbances to BBK32(51–80) were significantly higher in patients with early or late Lyme disease than in healthy control patients from a region of Lyme disease nonendemicity (*P* < 0.0001, EM+ or Lyme arthritis versus nonendemic healthy) or endemicity (*P* < 0.0001, EM+ versus endemic healthy; *P* < 0.005, Lyme arthritis versus endemic healthy), though not compared to patients with rheumatoid arthritis (RA) or syphilis (Fig. 2b). We observed positive antibody binding to peptide in 42/126 EM+ patients (33.3%) and equivocal antibody binding in 19/126 EM+ patients (15.1%) (Table 1). Positive and equivocal antibody binding to BBK32(51–80) in patients with Lyme arthritis was 3/20 (15.0%) and 2/20 (10.0%), respectively (Table 1). However, positive (Pos.) and equivocal (Eq.) antibody binding was also observed in serum samples from patients with RA (Pos., 4/42 [9.5%]; Eq., 6/42 [14.3%]) and syphilis (Pos., 5/31 [16.1%]; Eq., 4/31 [12.9%]) and in healthy volunteers (Pos., 2/101 [2.0%]; Eq., 3/101 [3%]) (Table 1). In total, the specificity, sensitivity, positive predictive value, and negative predictive value of BBK32(51–80) were 33.3%, 94.7%, 79.2%, and 70.2%, respectively.

**TABLE 1:** Antibody binding to BBK32(51–80)

Serum type	BBK32(51–80) result (no. of samples/total no. of samples [%])		
	Positive	Equivocal	Negative
Early Lyme (EM+)	42/126 (33.3)^*a*^	19/126 (15.1)	65/126 (51.6)
Lyme arthritis	3/20 (15.0)	2/20 (10.0)	15/20 (75.0)
Nonendemic healthy	2/101 (2.0)^*a*^	3/101 (3.0)	96/101 (95.0)
Endemic healthy	0/35 (0.0)^*a*^	0/35 (0.0)	35/35 (100.0)
RA	4/42 (9.5)^*a*^	6/42 (14.3)	32/42 (76.2)
Syphilis	5/31 (16.1)	4/31 (12.9)	22/31 (71.0)

a *P* < 0.01, comparing antibody binding in early Lyme disease patient sera to antibody binding the indicated control patient sera.

**FIG 3:**
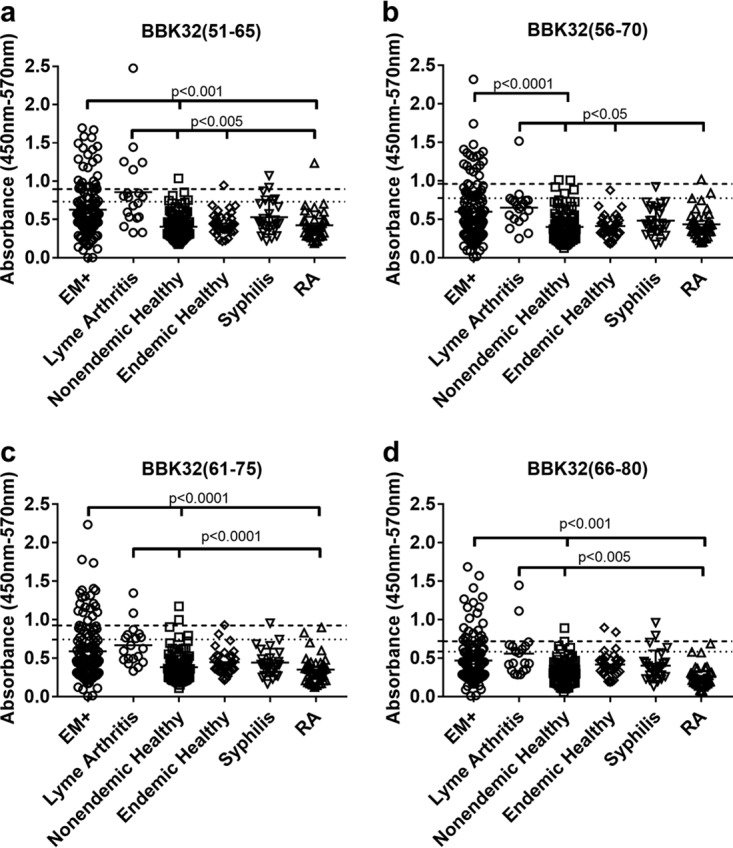
Antibody binding to BBK32(51–65) (a), BBK32(56–70) (b), BBK32(61–75) (c), or BBK32(65–80) (d) in serum samples from patients with early Lyme disease (erythema migrans [EM+]), Lyme arthritis, healthy controls from patients living in regions not endemic or endemic for Lyme disease (nonendemic normal and endemic normal, respectively), patients with syphilis, or patients with rheumatoid arthritis. The dashed line represents the cutoff for positive binding, 3SD from the mean (horizontal solid bar) of the healthy controls. The dotted line represents the cutoff for equivocal binding, 2SD from the mean of healthy controls. Antibody binding was measured by ELISA and the results presented as absorbance at nm 450 to reference absorbance at 570 nm. Total sample numbers (*n*) are indicated in Table 2. Comparisons were performed for all groups, and only differences that generated a *P* value of less than <0.05 are shown. All other comparisons are not significant.

Because most linear B cell epitopes are between 5 and 22 aa long (28), we hypothesized that BBK32(51–80) may contain more than one epitope. To test this, we evaluated antibody binding to the four overlapping peptides that comprised BBK32(51–80) in the epitope mapping to determine if antibody binding in serum from Lyme disease and control patients was associated with distinct regions within the larger 30-aa sequence (Fig. 3 and Table 2). As demonstrated in Table 2, we observed that nonspecific antibody binding, while reduced compared to that with the full BBK32(51–80) (Table 1 versus Table 2), was spread among the four component peptides (Fig. 3 and Table 2). Nonspecific antibody binding was not limited to or concentrated within a specific peptide. In addition, positive antibody binding in serum from EM+ patients to any of the component peptides was significantly reduced compared to BBK32(51–80) [(BBK32(51–80) versus BBK32(51–60), *P* < 0.05; BBK32(51–80) versus BBK32(56–65), *P* < 0.005; BBK32(51–80) versus BBK32(61–75), *P* < 0.01; BBK32(51–80) versus BBK32(66–80), *P* < 0.001] (Table 1 versus Table 2). These data strongly suggest that multiple epitopes contribute to the positive detection of both specific and nonspecific antibody binding in different individuals.

**TABLE 2:** Antibody binding to BBK32(51–80) component peptides

Serum type	Binding result (no. of samples/total no. of samples [%]) for:											
	BBK32(51–65)			BBK32(56–70)			BBK32(61–75)			BBK32(66–80)		
	Positive	Equivocal	Negative	Positive	Equivocal	Negative	Positive	Equivocal	Negative	Positive	Equivocal	Negative
Early Lyme (EM+)	25/109 (22.9)^*a*^	9/109 (8.3)	75/109 (68.8)	17/109 (15.6)^*b*^	13/109 (11.9)	79/109 (72.5)	19/109 (17.4)^*b*^	12/109 (11.0)	78/109 (71.6)	18/109 (16.5)^*b*^	8/109 (7.3)	83/109 (76.2)
Lyme arthritis	5/19 (26.3)	5/19 (26.3)	9/19 (47.4)	1/19 (5.3)	1/19 (5.3)	17/19 (89.5)	2/19 (10.6)	6/19 (31.6)	11/19 (57.9)	3/19 (15.8)	6/19 (31.6)	10/19 (52.6)
Nonendemic healthy	1/100 (1.0)^*a*^	4/100 (4.0)	95/100 (95.0)	2/100 (2.0)^*b*^	3/100 (3.0)	96/100 (95)	2/100 (2.0)^*b*^	3/100 (3.0)	96/100 (95)	1/100 (1.0)^*b*^	4/100 (4.0)	95/100 (95.0)
Endemic healthy	1/35 (2.9)^*a*^	0/35 (0.0)	34/35 (97.1)	0/35 (0.0)^*b*^	1/35 (2.9)	34/35 (97.1)	1/35 (2.9)^*b*^	1/35 (2.9)	33/35 (94.3)	2/35 (5.7)	2/35 (5.7)	31/35 (88.6)
RA	1/37 (2.7)^*a*^	0/37 (0.0)	36/37 (97.3)	1/37 (2.7)^*b*^	1/37 (2.7)	35/37 (94.6)	0/37 (0.0)^*b*^	3/37 (5.4)	35/37 (94.6)	0/37 (0.0)^*b*^	1/37 (2.7)	36/37 (97.3)
Syphilis	2/25 (8.0)	4/25 (16.0)	19/25 (76.0)	0/25 (0.0)^*b*^	1/25 (4.0)	24/25 (96.0)	1/25 (4.0)^*b*^	0/25 (0.0)	24/25 (96.0)	2/25 (8.0)	2/25 (8.0)	21/25 (84)

a *P* < 0.005, comparing antibody binding in early Lyme disease patient sera to antibody binding the indicated control patient sera.

b *P* < 0.05, comparing antibody binding in early Lyme disease patient sera to antibody binding the indicated control patient sera.

## DISCUSSION

**FIG 4:**
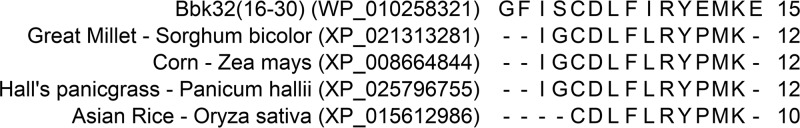
Sequence alignment of BBK32(16–30) between the B. burgdorferi B31 sequence used for epitope mapping (top row) and amino acid sequences in non-*Borrelia* spp. (excluding the genera *Borrelia* and *Borreliella* from analysis) identified using pBLAST. The sequence ID is the Latin name (NCBI RefSeq accession no.).

Previous studies demonstrated the diagnostic potential of BBK32; however, unacceptable levels of cross-reactivity were observed (22–26). In the present study, we evaluated linear B cell epitopes from BBK32 in an attempt to identify target sequences that could provide better specificity while attempting to maintain sensitivity. Two regions were identified by epitope mapping, BBK32(16–30) and BBK32(51–80). In subsequent analysis, BBK32(16–30) demonstrated a rare “universal” cross-reactivity. We observed antibody binding by ELISA at absorbance levels that we typically associate with positive binding (8, 12–14, 29) in almost all serum samples evaluated, including early and late Lyme disease, healthy controls, and disease controls. This indicates that the source of antigenic stimulation against this sequence is likely commonly encountered in human populations. Further evaluation of the sequence using pBLAST indicated that a sequence found in a DICER homolog in several plant species, including Zea mays (corn), Sorghum bicolor (great millet), and Oryza sativa (rice), had 60% identity with BBK32(16–30) derived from B. burgdorferi B31. Nine amino acids in the sequence running from BBK32(18–29) were identical to that found in the B. burgdorferi sequence (Fig. 4). While this association cannot be proven to be the cause of the observed nonspecificity, the presence of a conserved sequence within very common dietary foods is notable. Interestingly, though cross-reactive antibody binding was observed in previous studies utilizing whole BBK32 protein, they did not report the very high level of cross-reactivity that we observed for BBK32(16–30). The reason for this is unclear; however, it is possible that this sequence is not surface exposed in the whole protein, as is the case for the C6 peptide derived from the VMP-like sequence E (VlsE) protein of B. burgdorferi (30). Analysis of surface accessibility using the Emini Surface Accessibility Prediction tool for B cell epitopes at the Immune Epitope Database (http://www.iedb.org/) suggests that the region of BBK32 containing aa 16 to 30 is in fact not surface exposed (data not shown).

BBK32(51–80) was far more effective as a diagnostic target than BBK32(16–30), as positive or equivocal binding was observed in 48.4% of EM+ patients and 25% of Lyme arthritis (LA) patients, demonstrating a clear ability of the peptide sequence to differentiate patients with Lyme disease from those that do not have it (Fig. 2 and Table 1). It is unclear why positive antibody binding was reduced in LA patients compared to EM+ patients, as LA patients typically have a more robust antibody response and there is no evidence of BBK32 being downregulated during infection. However, we have observed this phenomenon previously with linear peptides derived from OspC, DbpA, and DbpB (12, 14). It is possible that as the antibody response matures it is primarily directed against conformational epitopes, reducing the response to linear epitopes; however, this remains conjecture. Unfortunately, we did observe an unacceptable level of antibody binding to BBK32(51–80) in negative-control sera. We analyzed the overlapping peptides that comprise the 30-aa region to determine if this cross-reactive antibody binding was associated with a specific region of the peptide. Cross-reactivity was reduced in each of the four peptides compared to BBK32(51–80); however, positive antibody binding was also reduced, and the antibody binding was not associated with a particular epitope within the 30-aa region. Though linear B cell epitope mapping of the BBK32 antigen of B. burgdorferi identified epitopes that conferred enhanced specificity compared to previously reported specificities for whole BBK32 protein (22–25) and protein fragments (26) (94.7% versus 93% and 88%, respectively), this specificity was still unacceptably low compared to the specificity of the 2-tier assay (>99% [7]). The importance of high specificity in Lyme disease diagnostics cannot be overstated. In the absence of a clearly identifiable erythema migrans, the symptoms of Lyme disease are nonspecific (fatigue, myalgia, arthralgia, irritability, poor concentration, etc.). The high incidence of nonspecific symptoms in the general population makes clinical diagnosis challenging in these cases (10, 31). Even in regions of high endemicity, in the absence of well-defined objective abnormalities compatible with Lyme disease and no history of exposure, the pretest likelihood of Lyme disease is low (32). Consequently, in a patient who presents with only nonspecific complaints, negative serology is highly predictive that the patient does not have Lyme disease. However, given the low pretest likelihood of disease and the high incidence of nonspecific complaints in the general population, the positive predictive value of positive serology in the absence of objective clinical abnormalities is unacceptably low. When “low” specificity is factored in, the positive predictive value of the assay has virtually no value. A specificity of 94.7% may not be considered low for some assays where there is a high pretest likelihood of disease, but in the context of Lyme disease, where over 3 million tests are being ordered each year, this would translate into over 150,000 false-positive results. Though specificity for BBK32 peptides is marginally improved compared to protein fragments or whole protein, it remains unacceptably low in the laboratory diagnosis of Lyme disease. Note that this discussion of specificity only scratches the surface of the myriad of complexities associated with laboratory diagnostics for Lyme disease but remains a particularly important issue for the development of new tests.

**FIG 5:**
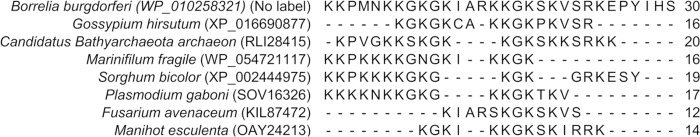
Sequence alignment of BBK32(51–80) between the B. burgdorferi B31 sequence used for epitope mapping (top row) and aa sequences in non-*Borrelia* spp. (excluding the genera *Borrelia* and *Borreliella* from analysis) identified using pBLAST. The sequence ID is the Latin name (NCBI RefSeq accession no.).

The functional aspects of BBK32 (extracellular matrix binding and complement binding) are shared by a large number of proteins across many prokaryotic and eukaryotic species. It is conceivable that antigenic structural elements and amino acid sequences would be shared among these proteins, though we did not identify specific proteins with high homology from other pathogenic bacteria in an expanded BLAST analysis. Further analysis of BBK32(51–80) sequence homology by pBLAST indicated some identity with a variety of antigens from different sources (Fig. 5). Though total homology was <50% for all sequences identified in the pBLAST search, homology was primarily associated with the numerous lysine residues found repeated through the BBK32(51–80) sequence. While not conclusive, this provides substantive evidence for a source of the observed cross-reactivity. In our experience, BLAST analysis alone is typically not an effective measure of potential cross-reactivity because conserved amino acids located within a potential epitope sequence may not contribute to antibody binding and can be irrelevant nonfunctional elements of the sequence. Therefore, actual screening of peptides using patient serum is required. This study highlights some of the difficulties in identifying sensitive and specific antigens that will be effective as serodiagnostic targets. In conclusion, we were unable to fully resolve issues of nonspecificity associated with BBK32 through the use of epitope-containing peptides as target antigens for serodiagnosis of Lyme disease. Care should be taken when using this antigen and many of the other B. burgdorferi antigens as targets because of the presence of cross-reactive epitopes contained within many of these antigens and the increased likelihood of generating false-positive results.

## MATERIALS AND METHODS

### Patient samples.

We performed a retrospective analysis using serum samples previously collected and stored at −80°C. Samples were from early and late Lyme disease patients obtained at Lyme disease clinics at New York Medical College in Westchester County, NY (*n = *68), Stony Brook University in Long Island, NY (*n = *20), and Gundersen Lutheran Medical Center in La Crosse, WI (*n = *57). Early Lyme disease serum samples (EM+) (*n = *126) were collected from patients presenting with one or more erythema migrans lesions, the distinct clinical marker of Borrelia burgdorferi infection. Serum samples from Lyme arthritis patients (*n = *20) were collected from patients upon first clinical presentation to the Gundersen Lutheran Medical Center in La Crosse, WI; diagnosis was made based on both clinical presentation (all had joint swelling) and a positive 2-tier serology result. All collection sites are in regions that are hyperendemic for Lyme disease, and the early disease sera were obtained during the height of tick-borne disease transmission in each region, which runs from mid-spring to mid-fall. Serum samples collected at New York Medical College were previously generously provided by Mary Petzke, serum samples collected from Stony Brook University were previously collected by Raymond Dattwyler, and serum samples from Gundersen Lutheran Medical Center were generously provided by Steven Callister. Samples were deidentified and delinked before being provided to us. Serum samples collected from healthy volunteers residing in regions not endemic for Lyme disease (New Mexico [*n = *61] and southern California [*n = *40]) were purchased from Creative Testing Solutions (Tempe, AZ) and Bioreclamation, LLC (Westbury, NY), respectively. Healthy control sera (*n = *35) were also collected from healthy individuals working at the Fire Island National Seashore, an area endemic for Lyme disease, as part of a Lyme disease surveillance study in Long Island, NY (through Stony Brook University) (33). Serum samples obtained from patients with rheumatoid arthritis (rheumatoid factor [RF] status unknown; *n = *42) or syphilis (rapid plasma reagin positive [RPR+] and antitreponemal antibody positive [ab+]; *n = *31) were purchased from Bioreclamation, LLC. These sera were sourced from the northeastern United States. Rheumatoid arthritis patients represent a well-defined group of control patients with elevated antibody and joint damage, which can occur in Lyme disease. Syphilis patients represent a well-defined group of individuals infected with a related spirochete. All serum samples used in this study were collected with consent under institutional review board (IRB) approval from the relevant institutions. Some serum samples were fully consumed during testing. As a result, some peptides were tested using fewer serum samples. Total sample numbers are indicated in Tables 1 and 2 for each experiment.

### Peptides.

Linear B cell epitope mapping was conducted by Proimmune, Ltd. (Oxford, UK) using their proprietary ProArray Ultra custom peptide microarray technology, as previously described (14). Overlapping peptide libraries consisting of 15-aa-long peptides, overlapping by 10 aa (5-aa offset) were generated using the sequence of BBK32 derived from the B31 strain of B. burgdorferi (NCBI RefSeq accession no. WP_010258321.1). The library was probed with eight serum samples from patients diagnosed with early Lyme disease that demonstrated strong antibody responses, defined as 9 to 10 dark bands on a commercial IgG Western blot analysis (Viralab, Inc., Rochester, MN). Synthetic peptides containing sequences of interest (see above) were synthesized by LifeTein, LLC (Somerset, NJ).

### Sequence analysis.

Sequence analysis was performed using the pBLAST algorithm at the NCBI (27). The pBLAST algorithm was used to identify conservation among Lyme disease-causing *Borrelia* spp. by restricting analysis to taxid: 64895 (Lyme disease *Borrelia*/Borreliella). Analysis of the epitope sequence in non-*Borrelia* spp. was performed by using the pBLAST algorithm, excluding taxid: 64895 (Lyme disease *Borrelia*/Borreliella) and taxid: 138 (*Borrelia*/relapsing fever-causing *Borrelia* spp.). Parameters were automatically adjusted to search for a short input sequence. Alignments were constructed using CLC Main Workbench 8.1 (Qiagen).

### ELISA.

ELISA was carried out using standard techniques, as previously described (14), using the following parameters: peptide coating concentration, 10 µg/ml in 10 mM sodium carbonate buffer (pH 9.4); blocking buffer, 1% bovine serum albumin (BSA) in phosphate-buffered saline (PBS); detection antibody, 1:15,000 dilution of horseradish peroxidase (HRP)-labeled goat anti-human IgM, IgG, and IgA (Jackson Immunoresearch, West Grove, PA); and substrate, SureBlue 3,3′,5,5′-tetramethylbenzidine (TMB) peroxidase substrate (SeraCare, Milford, MA). The colorimetric reaction was stopped using 2 N sulfuric acid, and absorbance was measured at an optical density at 450 nm (OD_450_) and OD_570_ using a SpectraMax Plus384 spectrophotometer. Absorbance data are presented as OD_450_ − OD_570_.

### Data analysis.

Statistical analysis was performed using Prism 7.0 (GraphPad, La Jolla, CA). Antibody binding absorbances were compared using a Kruskal-Wallis analysis of variance (ANOVA) followed by a Dunn’s multiple-comparison posttest. Categorical data were analyzed using a Fisher exact test. A *P* value of <0.05 was considered statistically significant.
